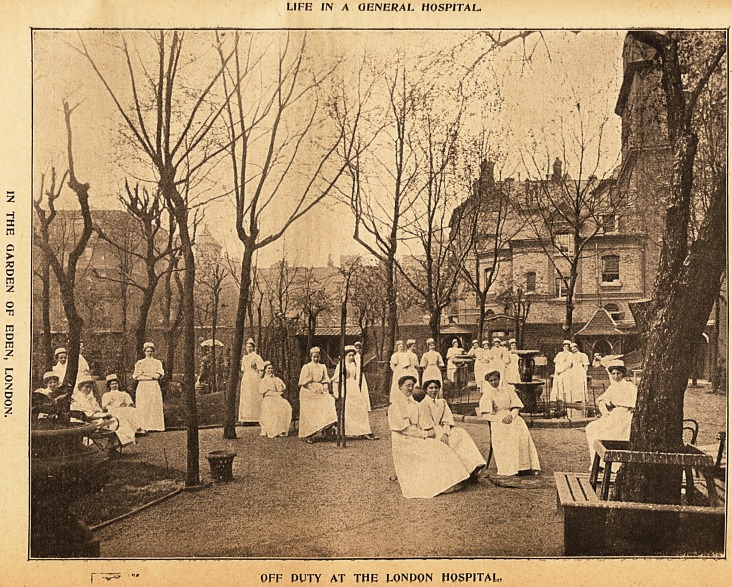# The Hospital. Nursing Section

**Published:** 1907-03-02

**Authors:** 


### The Hospital.

### Nursing Section.

Contributions for "THE HOSPITAL," should be addressed to the EDITOR, "THE HOSPITAL" NURSING SECTION, 28 & 29 Southampton Street, Strand, London, W.C.

No. 1,068.—VOL. XLI. SATURDAY, MARCH 2, 1907.

### Notes on News from the Nursing World.

### NURSES AND THE WRECK OF THE "BERLIN."

As soon as the wreck of the *Berlin* off the Hook of Holland was made known several nurses were despatched from Rotterdam to tend any sufferers who might need their services, and to prepare the dead for burial. The care bestowed by the nurses on the passengers in their terribly exhausted condition was admirable, but they were so affected by the sight of their patients that they found it difficult to refrain from tears. At the piteous request of a young girl in the crowd, who found it impossible to get any definite information, one of the nurses made inquiries for her missing relative, and had to break the news that he was not among the rescued. On Saturday the Amerika Hotel was besieged by people anxious to obtain a glimpse of the survivors, and vigorous measures were required to secure for the medical men and nursing staff the privacy and quiet needed for the convalescence of their patients.

### LORD ROBERTS ON THE WORTH OF NURSES.

The members of the Ascot Benefit Nursing Association had the pleasure last Friday of hearing a speech from Field-Marshal Lord Roberts, who affirmed that "his knowledge of the worth of nurses in South Africa was enough to fill him with gratitude." He remembered the time when there were no nurses in South Africa, and when antiseptic treatment and the use of anæsthetics were practically unknown. Contrasting his Indian experiences with those in South Africa, he said that for the first two or three months of the siege of Delhi, not a single case of amputation survived, and at Lucknow the death statistics were very similar. In fact, it would hardly be possible to describe what the wounded and sick suffered during the Indian campaign from the overpowering heat, the swarm of insects, the stench, the lack of surgical and medical treatment, and the want of proper nursing. During the South African War a totally different state of affairs prevailed, and the presence of trained nurses materially contributed to the difference.

### NURSING QUESTIONS AT THE POOR-LAW CONFERENCE.

At the Central Poor-law Conference in London last week, the question of the nursing of the outdoor poor and the administration of the Midwives Act in rural districts was discussed. It was introduced by Mrs. Charles Hobhouse, Hon. Secretary of the Wiltshire County Nursing Association, who, as to the first point, urged that the simplest and cheapest method of meeting the demands of relieving officers for nurses for the outdoor poor is for Guardians to subscribe to any nursing associations in their Union; and as to the second, that unless action be forthwith taken by public bodies or voluntary effort to provide a sufficient supply of competent midwives, Guardians will be called upon to pay extremely heavily for the services of medical men to meet the requirements of the Act. Subsequently, Dr. J. M. Rhodes moved, that in the opinion of the Conference, the Local Government Board should at once take steps to secure the licensing of competent Union hospitals to train midwives so as to meet the demand that at present exists, "all midwives to pass the same examination." This resolution was carried unanimously. We are glad that Dr. Rhodes recognises the importance of maintaining one standard for midwives.

### OVERWORKED NURSES AT SWANSEA GENERAL HOSPITAL.

There was a long discussion last week at the meeting of the Swansea Hospital Board on a proposal by the House Committee to appoint three additional probationers. In support of the proposal, Colonel Morgan mentioned that since August not one of the night nurses had had a single day's holiday, and the matron had told him that she could not release them for some time yet; while the Chairman, Mr. D. W. Hughes, declared that he would rather face the music at the Council meeting for increasing the expenditure than explain to the community why they were over-working the nurses. The report of the matron was then read, and she stated that the staff had been inadequate for some time; that the monthly day off or night off had frequently not been given; and that she was still being called upon to give last year's holidays. We are glad that it was determined to augment the staff as proposed. But the addition ought clearly to have been made earlier. No hospital of repute should expose itself to such a damaging admission as that of the Chairman at Swansea, who, in his speech, confessed that the authorities had been guilty of "sweating," and said that he was "actually ashamed of himself."

### A WISE DECISION AT NORTHAMPTON.

At the annual meeting of the Northamptonshire Town and County Nursing Association held on

Thursday last week it was announced that the Committee of the Queen Victoria Nurses' Home have resolved, after many meetings and much discussion, to relinquish private nursing in connection with the Institute. The Chairman, in making this statement, said that the Committee found that the district work of the Association had become so large that it was quite impossible to carry on any private nursing. There was an opportunity to hand over the latter to the General Hospital, and they had come to the determination to avail themselves of it. We are sure that a very wise decision has been arrived at. The report for 1906, which was adopted, shows the extent of the district work, and also that it is increasing. The number of cases nursed in 1906 was 601, as against 565 in 1905; and the number of visits paid was 18,017, as against 16,466. It will readily be understood, as the Chairman indicated, that to arrange the private nursing in addition has involved great responsibility and much trouble. We entirely share his opinion that it is better to concentrate attention on the district work, especially as it is intended to extend it to maternity nursing, for which there is a great need. We always regard the combination of private with district nursing as a mistaken policy, and the abandonment of a system which has admittedly broken down at Northampton is a matter for congratulation.

### ALIENATING PUBLIC SUPPORT.

The Bishop Auckland Guardians have some reason for declining to contribute to the District Nursing Association, if, as "A Twenty Years' Resident" assured us last December, the committee require the nurses to attend the wealthy for the reduced fee of 15s. A long interval has elapsed since this statement was made, but we have not received any contradiction, although we invited one; and the fact that in the annual report the words "sick poor" are omitted, tends to confirm the allegations that the mistaken policy of employing public subscriptions for the purpose of securing cheap nursing for the well-to-do is being pursued. It is not surprising that District Nursing Associations which expose themselves to criticism do not enlist the maximum of outside sympathy. A difficulty has now arisen in connection with the Wolsingham Association, which is in open conflict with its nurse. The nurse, it appears, is accused of prejudicing the patients of a medical man and of questioning his treatment, with the result that her resignation has been demanded. She has, however, a number of supporters in the district, and they threaten to elect a council who will re-engage her. In the interests of the nurse we believe that this would prove a regrettable step for her supporters to take. The success of district nursing depends so entirely on amicable relations being established between the medical men and nurses, that when friction arises, as may happen without much fault on either side, unless the subscribers can adjust matters, the only dignified course for the nurse to pursue is to waive the whole matter in dispute and take up work elsewhere under more congenial conditions.

### EXETER GUARDIANS AND NURSES.

At a meeting of the St. Thomas (Exeter) Board of Guardians last week it was reported by the Visiting Committee that, in accordance with resolutions passed by the Guardians at the last meeting, the clerk had submitted a statement of the changes in the nursing staff since January 1, 1905. The Committee recommended the Guardians to appoint a superintendent nurse instead of a head nurse for the sick wards, such superintendent nurse to have full charge of the sick inmates and the supervision of the assistant nurses, without any direction from the matron. The salary of the superintendent nurse to be fixed at £34, rising by yearly increments of £1, to £38 per annum. The report was adopted. At a previous meeting the Guardians declined to allow an assistant nurse who had obtained another appointment to leave without notice, as it would be a bad precedent to establish. It was reported that the nurse in question had paid a month's salary in lieu of notice and left the institution.

### IMPERIAL MILITARY NURSING SERVICE.

Miss R. Beamish and Miss C. E. A. Harries have been appointed staff nurses in Queen Alexandra's Imperial Military Nursing Service; Miss B. M. Nye has been appointed to the Royal Herbert Hospital, Woolwich, instead of the Royal Victoria Hospital, Netley; Miss M. C. Watson, to the Royal Herbert Hospital, Woolwich; Miss M. H. Smyth and Miss E. McGrath, to the Millbank Hospital, London; and Miss R. Bramish, to the Cambridge Hospital, Aldershot. Miss K. Coxon has been transferred to the Military Hospital, Portsmouth, from the Cambridge Hospital, Aldershot; and Miss M. A. Cachemaille, to the Cambridge Hospital, Aldershot, from the Military Hospital, Portsmouth. The appointments of Miss V. L. Batteson, Miss A. M. Clapp, Miss M. H. Congleton, Miss A. R. Sibbald, and Miss H. Winzer have been confirmed. Sisters B. S. Vaughan and C. K. E. Steel have arrived from South Africa.

### THE QUESTION OF PASSES.

At the last meeting of the Keighley Guardians it was proposed to abolish the passes. One of the Guardians said he thought that it was humiliating for a nurse to have to make application to the matron when it was "her day out"; and in order to illustrate the waste of time and money, he mentioned that in a single fortnight no fewer than a hundred passes had been required. He described the system as tyrannical. The only defence of it is that it was introduced five or six years ago owing to irregularities. The matter was ultimately referred to the Infirmary Committee to discuss and report upon to the Board. In a well-managed institution passes are not necessary, and in one that is not well managed we doubt whether they are of any practical value. They cause irritation without ensuring a compensating advantage.

### MISS NOTT-BOWER AT GUY'S HOSPITAL.

On Wednesday evening last week Guy's Hospital Nursing Society held an interesting meeting in the Court Room. The chair was taken by the Chaplain, the speaker was Miss Nott-Bower, a former matron of Guy's. She gave a very graphic and comprehensive account of her work among the navvies of Grahamstown, South Africa, which she took up soon after she left London in 1900. She spoke of the difficulties and charms of the nursing and mentioned that the caravan locomotive, which is the chief method of travelling, is attachable to trains and is conveyed free, so that the nurses may reach the very scattered homes of the navvies. The caravan is often left on a siding and forms the nucleus of a district. Miss Nott-Bower said that more volunteer mission nurses amongst these people are much needed. The collection was for the funds of the Navvy Mission.

### THE RECOVERY OF MISS LOWE.

We are glad to learn that Miss Lowe, the member of the Nurses' Co-operation who was murderously attacked in the Mont Cenis Tunnel a few weeks ago, was discharged from the hospital at Chambery on Sunday. She has quite recovered, and left at once for Paris in company with the nurse who was despatched from New Cavendish Street to attend upon her. At the outset the friends of Miss Lowe were afraid that the injuries inflicted by her assailant might have serious results, but these forebodings have happily not been verified, owing largely, no doubt, to the skill of the medical man and the care with which she has been nursed.

### ROYAL HANTS COUNTY HOSPITAL.

At a meeting of the Governors of the Royal Hants County Hospital last month, the proposed new block for nurses was discussed. The block, upon which a sum of £3,000 is to be expended, will consist of bicycle, linen, and cloak rooms, a store, sitting-room, sisters' bed-sitting-room, drying-room, and bathroom. The laying of the foundation-stone of the block is to be made the occasion of an interesting function.

### NURSES AND THE MOTHERS' UNION.

In the first annual report of the Sub-Committee of the Mothers' Union on the work among nurses it is stated that, after the initial drawing-room meeting at Camelford House in support of the movement, addresses by diocesan speakers on its behalf were given at Guy's Hospital, St. George's, the Westminster, the Samaritan Free, the Orthopædic, Plaistow Maternity Hospital, and several other institutions; also to district nurses from a number of centres. In every case the nurses have expressed themselves in complete sympathy and willing to do all they could to further the aims and objects of the Mothers' Union. Many from different parts of the country have written offering their co-operation, and one nurse in South Wales distributed over 500 leaflets. Two new branches have been the outcome of the energetic work of nurses, and in one instance the matron and five nurses in a maternity home have become associates; and, as the result of holding regular meetings for three months in the Home, thirty-five former patients have been enrolled as members. The matron of this Home writes that "the improvement in character and effort in these mothers is very marked."

### SCARCITY OF TRAINED MIDWIVES.

Leicestershire is suffering from a scarcity of certificated midwives, and the medical officer of health for the county has sent a circular letter to all the local boards of guardians and rural district councils on the subject. The Education Committee of the Leicestershire County Council award scholarships for the training of midwives, and the medical officer of health asks boards of guardians and district councils to supply him with the names of suitable women to take up the work.

### NURSES' SOCIAL UNION.

By invitation of Mrs. Daubeny a meeting of the Nurses' Social Union was held at her house in Bath on Friday last. The lecture was given by Mr. Frederick Lace, F.R.C.S., L.R.C.P., on "The Newer Methods of Nursing after an Operation." Miss Eden, central organiser, also spoke to the nurses on the development of the movement. Between sixty and seventy nurses were present. There will be a meeting at Taunton on March 6, and another at Minehead on March 20.

### FUND FOR NURSES IN IRELAND.

At a special meeting of the Council of the organisation known as King Edward's Fund for Nurses in Ireland last week the resignation of Miss M. E. MacDonnell, the Secretary, who is going abroad, was announced, and it was decided to make a further grant of £10 to a nurse member who had received a similar amount in 1906, in order to help her over a tedious convalescence after an operation.

### CLAIM AND COUNTER-CLAIM.

At the Sussex Winter Assizes last week two cases were heard of alleged slander, the plaintiff in one being a nurse and the defendant a medical man; while in the second action the positions were reversed. Both plaintiffs got a verdict, but the nurse only received a farthing damages, while the medical man was awarded £25, Mr. Justice Bucknill, the Judge, remarking that he entirely agreed with the findings.

### WALLINGFORD COTTAGE HOSPITAL.

The post of matron of Wallingford Cottage Hospital recently became vacant by the resignation of Miss Ashley. But after the vacancy was advertised Miss Ashley withdrew her resignation by consent of the committee, and no change has therefore been made.

### SHORT ITEMS.

At a meeting of the Council of the Queen Victoria Jubilee Institute for Nurses on Thursday last week Viscount Goschen was re-elected Chairman of the Council.—An inquest was held last week on the body of Mrs. Anna Sophia Harper, a nurse who, according to the finding of the jury, threw herself over the cliff at Rottingdean during a state of temporary insanity.

### The Nursing Outlook.

"From magnanimity, all fears above;
From nobler recompense, above applause,
Which owes to man's short outlook all its charm."

### MAKE-BELIEVE IN BRITISH NURSING.

#### III. THE STAGE ARMY MUST FACE THE FACTS.

We have shown that the Matrons' Council, though it is only a small, non-representative body, is, in fact, the mother and also the bone and sinew of the International Council of Nurses, of the Society for the State Registration of Nurses, and of the National Council of Nurses, if, indeed, the latter exists. These facts are important and they must be faced by the Stage Army.

We recognise that an International Council of Nurses, organised on a representative basis and embracing the leaders of nursing in all civilised countries, would be a desirable thing in itself and might fulfil many useful purposes. But at present there is no such International Council of Nurses. The few ambitious women, who have taken, or been given that name by the Matrons' Council, cannot claim any representative character. This so-called International Council should, therefore, be speedily ended or mended, if the real interests of the nursing profession are to be the paramount consideration. We are merely upholding the highest interests of the profession and voicing the best British nursing opinion by making these facts plain, for we thus prevent any misapprehension as to the actual present position. It is widely felt to be impossible to permit the creation of an oligarchy in British nursing. The leaders of the present attempt have already proved mischievous by blocking the way to all joint action, to all national courtesy, and to all unity of aim in nursing affairs. All the independent leaders of British Nursing will endorse this opinion. For ourselves, we have nothing to do with individuals as such. It is the principles which matter, for without principles no organisation, for any public or professional purpose, can ever prove successful.

The absurdities of the present position are well illustrated by the paper which calls itself the Official Organ in the British Empire, of the Matrons' Council, of the International Council of Nurses, and of the Society for the State Registration of Trained Nurses. This "Official Organ," instead of attempting to meet the facts which we have made clear in our two former articles, merely inserts a violent, and, indeed, one might say a vulgar attack, in the course of which the following words occur: "They (*i.e.*, 'Professionally minded nurses'), prefer" this 'Official Organ' "owned, managed, and edited by trained nurses for the benefit of nurses, and small blame to them."

Amongst the articles which appear in the same issue of the "Official Organ" in which this laudatory notice is published is one entitled "A Lay History of Nursing," from which the following are extracts:—

"The *American Journal of Nursing* sums up Mrs. Sarah Tooley's History of Nursing in the following pithy paragraph:—

"'There are two ways of writing history, one of putting things in, and another by leaving them out. . . . The truth is, the woman who has been the foremost and fearless leader of this movement (*i.e.*, 'association and union among nurses for high purposes') in Great Britain, Mrs. Bedford Fenwick. . . .

"'. . . The whole splendid edifice of constructive work in education, organisation, civic activity, practical nursing reforms, training school progress, sound and honourable industrial conditions for nurses, and the development of an intelligent and ethical nursing press, which has been built up with distinguished ability by Mrs. Fenwick,'" (and four or five other ladies whose names are given) "'. . . must necessarily be left unnoticed and unsung.'"

The review from which the foregoing is an excerpt appears in the Foreign Department of the *American Journal of Nursing*, which is in charge of Miss Lavinia L. Dock. It will be remembered that Miss Dock is an honorary member of the Matrons' Council and also Secretary to the so-called International Council of Nurses, of which Mrs. Bedford Fenwick is either a President or the ruling spirit.

But again, the *American Journal of Nursing* appears also in another place in the same issue of the "Official Organ" which "professionally minded nurses" are said to prefer. Thus we read, under the heading of the "Paris Conference," "Mrs. Bedford Fenwick will open the Session at the Paris Conference on Nursing Organisation and the place of Professional Journals therein, and Miss M. E. P. Davis, of Boston, U.S.A., who was the actual promoter, and who took a leading part in founding the *American Journal of Nursing*, will sketch its history."

The uninstructed layman may well ask, "Who is Mrs. Bedford Fenwick whose praises are sounded by the *American Journal of Nursing*, with the promoter of which paper she is to appear at the Paris Conference?" Here, he will say, is a lady to whom the merits of the "Official Organ," as described by itself, will indeed appeal, and whom the editor of that paper may well hold up as a model to the "professionally minded nurse"! By turning to the first page of the "Official Organ" he will find the information he desires. He will learn—perhaps with surprise—that Mrs. Bedford Fenwick is herself the editor of the very paper (*i.e.*, the "Official Organ") from which the above extracts are taken. He will probably reflect that in the circumstances further comment is unnecessary.

### The Care and Nursing of the Insane.

By Percy J. Baily, M.B., C.M.Edin., Medical Superintendent of Hanwell Asylum.

### II.—NURSING THE SICK.

(*Continued from page* 291.)

3. *Nutrient Enemata.*—Although the mucous membrane of the lower bowel is capable of absorbing fluids, it contains no glands whose secretion has any digestive power. It follows, therefore, that any nourishment administered through this channel must be artificially digested before it is injected into the rectum. This can be readily done by means of various preparations of pancreatic ferment that are on the market, and whose method of use will be explained when we speak of sick-feeding. The bowel is not very tolerant of food, even if it be thoroughly predigested by artificial means, and this kind of enema is, if its use be continued for any length of time, very apt to set up irritation of the mucous membrane of the rectum, and consequent diarrhœa, which cannot be avoided unless by the constant care of the nurse. The absorption of fluids in the rectum is very slow and limited, and therefore the amount given at each injection must be small—as a rule about two ounces is sufficient, and the amount must never exceed three, or at the outside, four ounces. If the rectal feeding is to be continued the smaller amount mentioned should not be exceeded. The interval which elapses between each administration must be at least six hours. This, however, as well as the size and composition of the enema, is a matter which will, of course, be decided by the medical officer in charge of the case. A copious purgative enema of soap and water should always precede the first nutrient one, otherwise there is a risk that the contents of the lower end of the large bowel may be discharged into the rectum, which in health is always empty, as soon as the nutrient enema is injected into it, rendering it impossible for it to be retained. Moreover, when this form of feeding has to be depended upon for many days, as in the cases of ulcer of the stomach with hæmorrhage, for example, the purgative enema should be occasionally repeated, or every second day the bowel may be washed out with 1½ or 2 pints of saline solution (ʒij of table salt to one pint of warm water). This is to be administered in the same manner as an ordinary purgative enema, and the patient should be directed to expel it in the course of a few minutes; or the bowel may be washed out before each injection with saline solution or boracic acid lotion in the manner to be afterwards described. All these precautions are necessary in order to prevent the bowel from becoming irritable and rejecting the nourishment at once. For the same purpose also a few drops of laudanum —ɱv. to ɱx., according to instructions, may be added to every third enema. For general use the enema may consist of 1½ oz. milk with one new laid egg, which should be well beaten up before being mixed with the milk. After this has been thoroughly digested, one or two tablespoonfuls of warm brandy or port wine may be added.

The best apparatus to use for injecting the enema consists of the barrel of a two-ounce glass syringe, to which a rubber nasal feeding tube of small calibre is attached. The end of this tube is to be inserted into the anus for two or three inches, as in the administration of an ordinary enema, the buttocks of a patient being well propped up on pillows; the barrel of the syringe is then to be held up as high as the length of the tube will permit, and the prepared enema poured into it, when it will slowly run through. If the bowel is to be washed out, before such injection as suggested above the apparatus is to be first filled with the saline or boracic acid solution warmed to 95°. As the fluid in the barrel gradually sinks it is lowered until, by the time it is empty, it is level with the patient's buttocks; it is then inserted into a vessel and placed well below the bed, when the fluid will return from the bowel through the apparatus into the vessel. When all the fluid has then run out of the bowel the process may be repeated, or as the fluid gradually sinks more may be poured into the barrel before it is lowered, so as to wash out the rectum with more than the two ounces of fluid which the barrel holds, but not more than 6 ounces (three barrels full) should be used. When the washing out is then completed the nutrient enema is to be given. During the washing out the buttocks of the patient should not be raised. The object of raising the buttocks is that the fluid of the enema may tend to flow away from the anal orifice, and thus diminish the risks of its expulsion. When all the enema has run through the tube is to be gently withdrawn from the anus and the patient told to lie quietly for some time without changing his position.

### 7. Hot and Cold Applications.

*Cold Applications* in the shape of compresses or ice-bags are occasionally used where it is desired to diminish the amount of blood in a part, as during the early stages of inflammation. The cold stimulates the involuntary muscular fibres which are found in the walls of the small blood-vessels, and thus diminishes their calibre and the amount of blood passing through them. Cold compresses should consist of three or four layers of flannel or lint wrung out of iced water. These are then applied to the part and covered with a piece of jaconet or gutta-percha tissue. They require to be frequently changed, otherwise they become hot and act as poultices, and are then worse than useless for the effect for which they are ordered. Ice-bags made of india-rubber may be used for the same purpose. The ice should be broken up into small pieces by means of an ice-breaker, or if such a thing is not at hand it can be easily broken by pressing the point of a large needle into it with a thimble. The water which collects in the bag from the melting of the ice must be frequently poured out, and fresh pieces of ice put in to replace that which has thus disappeared.

*Hot Applications* are either moist or dry. The former include poultices and fomentations or stupes. Hot applications, in whatever form they are used, have an effect on the blood-vessels of the part to which they are applied which is the opposite of that of cold. That is to say, they cause the smaller arteries to dilate, and thus allow of a greater amount of blood to pass through. In this respect moist applications are very much more efficacious than dry ones, and in addition they tend to soften and relax the tissues and relieve spasm and pain. Whenever a nurse is about to apply any of these remedies she must be careful not to burn the patient. The poultice or fomentation should be tested against her own face before it is placed against the patient's skin. This precaution is doubly necessary when the patient's vitality is much lowered by disease, especially when there is dropsy or any kind of paralysis or other nervous disease where the sensibility of the patient's skin may be diminished. In such patients the heat which an ordinary person could bear with comfort may be sufficient to cause burns and consequent wounds which are very slow to heal.

*Poultices.*—These may be made of any sort of meal or of starch. There is, however, nothing better than linseed, for this meal contains a considerable amount of oil, and retains the heat better than anything else, and this should always be used unless it cannot be procured. To make a linseed poultice it is necessary to have an ordinary pudding basin and a spoon, together with a piece of muslin, linen, or some tow on which to spread it. Place the spoon in the basin and pour some boiling water into it. Having thus heated the spoon and the basin, pour the water away; then pour into the basin as much boiling water as will be necessary to make the poultice of the required size, and into this with the left hand sprinkle the linseed meal, stirring vigourously all the time with the spoon. When the mixture has reached the proper consistence—it should not be too firm—and is free from all lumps turn it out into the muslin or tow and spread it evenly and quickly. The mass should be about half an inch thick—if thinner than this it soon loses its heat, and if thicker it is heavy and uncomfortable, and should be spread so as to leave at least an inch of the linen or tow all round the edges. This is then folded in over the poultice, which is now ready to apply to the patient. The poultice should, if possible, be made near the patient so as to avoid having to carry it far; but if it should have to carried it should be folded over and wrapped in a layer of cotton wadding or placed between two heated plates. When applied it should generally be put next to the skin without any intervening material; if properly made it ought not to stick to the patient's skin; in any case this may be prevented by spreading a teaspoonful of heated olive oil over the poultice as soon as it is made. After the poultice has been placed in position it should be covered with a layer of cotton wool and then tied on with gauze bandages.

A linseed poultice will usually remain hot for three or four hours, and must never be left on the patient for longer than this time. It should always be changed as soon as it becomes cold or uncomfortable. When changed, the new one must be ready to apply before the old one is removed.

(*To be continued.*)

### The Nurses' Clinic.

### A CASE OF EMERGENCY.

Work was heavy and hands were few in a provincial hospital whither I had gone to take temporary duty for three months; in a word we were understaffed. I was at once the night nurse and night sister of the male side of the hospital. Accidents I also had to admit, there being no night porter, and, if necessary, prepare the theatre for operations. There was the usual busy, rapid routine of hospital work, only rather more so than usually falls to the lot of one nurse. I had no time to sit still and think enviously of those who were spending the night in the orthodox way and deplore the anomaly of night nursing. I was, at any rate, spared the subtle, insidious temptation of sleepiness which, in spite of the inevitable cup of tea, will assail the tired night nurse if she has not much to do.

"Nurse, will you get the isolation ward ready at once, please, for a bad case of diphtheria—a boy, seven years old—tracheotomy will be performed in the ward directly he arrives, in about an hour's time, and—will you take the case?"

It was the house surgeon who spoke. I had learnt the discipline of ready obedience to doctors' orders, so I replied in the affirmative, and then asked tentatively how my work was to be done. The best arrangement that could, under the circumstances, be made was made, and my place in the wards was taken by another nurse and I was left free for my new and responsible duty. There is no such thing really as monotony in hospital life, that word should never find a place in a nurse's vocabulary; it savours of lack of imagination and sympathy on her part, who would do well to remember that what is an "interesting case" to her, spells something very different to the object of her interest. Nevertheless an emergency case is the trained nurse's opportunity, and must not be discounted. But this in parenthesis. To get the fire lit, bed made, tent erected, kettle half-filled with boiling water, and put in motion, and to make, other preparations necessary for the operation, and for the nursing of such a case, did not take very long.

One glance at the poor little sufferer, who was brought in by his mother, convinced me that it was a very bad case; the child was *in extremis*, semi-suffocated by the cruel disease so often characterised by the appearance of membrane, of a more or less glutinous nature, which attacks and adheres to the throat and nasal passages. Fortunately the patient's arrival was soon followed by that of the operating surgeon and, all being in readiness, the operation was performed at once. The immediate result of the incision into the trachea was a rush of confined air, and with it a splutter of mucus, which I, standing too near, received into my face—carelessness for which I deserved no pity. The relief was instantaneous, and the look of strain and suffering on the little face was replaced by one of comparative comfort and ease. However, Johnny was in a critical condition, and I watched him anxiously for 18 hours, keeping the tube clear and giving constant nourishment, disinfecting and cleansing the throat, etc.

For that and the two following nights he did fairly well; in fact, speaking in the comparative degree, he did very well, and so I believed and hoped he would weather the storm, for he had that which is such a valuable asset in times of sickness—youth and strength. On the third night, or fourth—I cannot clearly remember which—all went well for the first few hours, his breathing and his strength well maintained. Suddenly, without any warning at all, there

THE NURSES' CLINIC.—*Continued.*

appeared to be an interruption in the breathing of a very serious nature, and poor little Johnny was threatened with suffocation, due, I rightly guessed, to a piece of membraneous matter having become dislodged from the lung, and blocking the tracheal passage beyond the reach of the tube. As long as I live I shall never forget that poor child's face; it was transfigured, his eyes, big with terror, were turned to me in agonised mute appeal, while he clenched his fists and kicked out his legs with the force of impotent frenzy. It was obvious that removing the tube would be of no avail, the tube was clear. For a moment my own helplessness was borne upon my mind with sickening dread. Must I watch the poor little fellow die!

My own agony of mind was as great as his physical distress. There was only one thing to be done, and if that failed, nothing could save the child—artificial respiration. I seized the arms and brought them above the head; in bringing them down to the sides I pressed with force against the ribs, to force the obstruction upwards if possible.

Hearing a nurse pass the door outside, I told her to call up the house surgeon immediately; he appeared almost at once.

"I can do nothing more than what you are doing, nurse," he said, and unwilling, I suppose, to watch what seemed to be inevitable, he, half-reluctantly, left the ward. Time cannot be measured in such supreme moments of life—it materialises itself to the overwrought brain, and merges into tangible torture.

Obviously, and as a matter of fact, no length of time can elapse in a case like this, so it must have been very shortly after the house surgeon left the ward that the child became slightly easier; soon he began to cough, and as he did so I caught sight of something appearing at the mouth of the tube, and was just about to seize it with a pair of forceps, holding my own breath in the extremity of my suspense, when, with inhalation, it disappeared down the tube again. However, the worst was over, the boy could draw his breath. I waited patiently with forceps in hand, watching the tube, as keenly as a cat watches a mouse-hole, and I have no doubt with a wildly-beating heart. Another cough and I had caught the thing, the cruel thing that had so nearly cost my little patient his life. A large glutinous piece of deadly membrane, about as large as the end of my finger, the largest piece I had ever seen. No sooner had the obstruction been coughed up than the child closed his eyes, breathed easily, and slept with all the anguish that had recently contorted his face, gone. I looked at the sleeping boy, and then at the thing I held in the forceps, and my eyes filled with tears—tears of joy, as the tension of my brain relaxed, and I realised that my efforts to save the child's life had not been in vain. I put it into a bottle containing methylated spirit, I held it up to the light, and looked at it again, with—oh! what different feelings. It was in the right place now, not in the wrong; that made all the difference. Now it was a bacteriological specimen! I looked at it with the keenest interest, almost with affection, for had it not negatively saved my dear little patient's life?

The house surgeon, who had not gone back to bed, returned just at the moment of my triumph; he was surprised to find the child sleeping and breathing normally, his extremity past, and no trace of it left on his features. I held the little bottle up to him with a smile on my face. He understood, gave an answering smile, and returned to bed. Easy respiration was established after that, and there was no recurrence of the impediment.

### Incidents in a Nurse's Life.

### MY FIRST MATERNITY PATIENT.

I was studying for my midwifery certificate—it was before the days of the Central Midwives Board—but before I could go to the hospital I had to attend a certain number of cases with a midwife or doctor. A friend of ours, a doctor living very near, said I should attend some cases with him. Accordingly a few days later he sent me to a case, arriving himself a few minutes afterwards.

I was only twenty-two and very nervous, but everything went off well, though I saw very little, as I kept on the other side of the room except when wanted by the doctor. The next week the doctor sent again to tell me to go to a certain address. I went and found a big buxom woman walking about and apparently in the first stage of labour. The doctor arrived shortly afterwards, and in a few minutes he said to me, "Well, nurse, she will be some hours yet" (this was about 10 A.M.), "I will go and see some of my patients and come back about noon." I felt quite a shiver run through me at the thought of being left alone, so I followed the doctor downstairs and begged of him not to be long. "Oh," he said, laughingly, "I shall be back long before you want me," so I returned to the bedroom and proceeded with my preparations and kept chatting with my patient. However, shortly I noticed that her pains were becoming stronger and at shorter intervals, and I began to feel increasingly nervous, and went to the window to see if I could see anything of the doctor's carriage, but there were no signs of him. At last the patient said, "I suppose you will be able to manage, nurse, if it comes before the doctor arrives?" My heart jumped into my mouth at this, but I said as bravely as possible, "Oh, yes," but it was a good thing she could not see my face. I went to the window again, and heartily wished I could jump through and run away. All at once the patient said, "Oh, nurse, I am sure it is coming." I went to her and found the head just emerging, and in two minutes a fine boy was born. I was trembling in every limb, and as to severing the cord I felt sure that the baby would bleed to death, or something awful would happen. But there was nothing to do but to go ahead, so I tied the two ligatures as well as I could, severed the cord, and rolled the kicking, bawling baby up in the receiver and put him in a place of safety. The third stage came off as easily as the rest had done, to my great relief. I then bound up the mother and made her comfortable. I bathed the baby and put him in bed with the mother. Presently I heard the sound of carriage-wheels, and then the doctor came striding up the stairs. "Well nurse," he said, "how are things by this?" "All right, doctor," I replied, he went to the bedside and the patient said, smilingly, "Well, you're a nice man, coming when all is over; look here," and she turned back the clothes to show him the baby, tugging away at the breast. The doctor did indeed look astonished, and smiled amusedly at me.

I told the mother all about it about two years afterwards when I was attending her again, and she is never tired of telling people that "their Tommy was Nurse G.'s first baby."

### Illustrations of the Life of a Modern Nurse.

LIFE IN A GENERAL HOSPITAL.

IN THE GARDEN OF EDEN, LONDON.

OFF DUTY AT THE LONDON HOSPITAL.

### Central Midwives Board.—February Examination.

COMPLETE LIST OF SUCCESSFUL CANDIDATES.

The number of candidates who presented themselves at the examination of the Central Midwives Board, on February 12, was 389, and of these 291 passed, the percentage of failures being 25.2. The following were the successful candidates:

Jane Bell, Janet Christiana Blomfield, Grace Edith Blott, Minnie Elsie Clark, Louise Mary Counsell, Lucy Dobson, Alice Kathleen Fenn, Rose Ethel Johnstone, Liza Irwin, Beatrice Llewellyn Margrave, Victoria Melicent Moore, Emma Polden Parks, Minnie Randell, Louisa Septima Robson, Charlotte Ann Tweedy, and Winifred Julia Woodforde, were trained at Queen Charlotte's Hospital.

Selina Amelia Adams, Mary Andrews, Carrie Caffin, Elizabeth Winifred Eady, Marion Gration, Evelyn Hastie, Jessie Judge, Emilie Sarah Lawrence, Annie Lewis, Grace Anna Josephine Lloyd, Katharine Vincent Lloyd, Neta Winifred Mackintosh, Olive Matthews, Lilian Helen Harrington O'Reilly, Amy Jane Pilton, Alice Muriel Ragg, Dorothy Rose Roberts, Rose Isabella Shephard, Edith Letitia Stephens, Emma Eliza Stiff, Frances Ellen Stillwell, Gertrude Ada Taylor, May Louisa Trenery, Daisy Maud Cottingham Wadling, Alice Wall, and Alice Maud Whitehead, at the General Lying-in Hospital.

Margaret Dorothea Palmer, at the City of London Lying-in Hospital.

Mary Archer Briggs, Jessie Crerar, Emily Alice Dibblin, Hilda Marjorie Edmonds, Emily Kezia Marle, Florence Victoria Earish Munson, and Alice Oliver, at Guy's Institution.

Gertrude Fanny Burnell, Alice Maud Elliot, Charlotte Sarah Elsey, Charlotte Elizabeth Ann Glossop, Mary Jane Houlton, Rose Anne Lloyd, Frances Drummond McGregor, Isabella Margaret Louisa Manners, Grace Anne Payne, Ada Mary Smith, and Mary Isabel Wigham, at the London Hospital.

Ellen Beadon, Catherine Bone, Caroline Eliza Green, Catherine Hester Harper, Amelia Blamey Northcott, Hélène Ullmann, and Constance Watney, at the Clapham Maternity Hospital.

Anne Sylvia Parker and Helen Edith Augusta Tottenham, at the New Hospital for Women.

Alice Barter Barter, Elizabeth Besgrove, Agnes Olive Blacklocks, Elaine Margaret Brewin, Rosa Bullock, Ella Ermyntrude Dixon, Charlotte Evans, Mary Jane Giddings, Helen Elizabeth Grahame, Margaret Gray, Mary Ann Grier, Laura Beatrice Gumbrell, Mary Harriet Helen Hill, Ellen Kneebone; Annie Macqueen, Annie Mary Meagrove, Martha Meredith, Millicent Rose Page, Elizabeth Pick, Minnie Pope, Margaret Annie Smith, Lizzie Taylor, Charlotte Turnbull, and Esther Elizabeth Welch, at the Maternity Charity, Plaistow.

Ethel Margaret Collins, Jane Edith Highet, Clara Reeve, and Olive May Pounds, at the East End Mothers' Home.

Emily Gooch, Lizzie Parker, Martha Elizabeth Pollexfen, Mary Elizabeth Wilkinson, and Ethel Womald, at the Salvation Army Maternity Hospital.

Margaret Hannah Hassell and Annie Paterson Wilson, at the Military Families' Hospital, Cheltenham.

Hariot Evans, at the Military Families' Hospital, Woolwich.

Nettie Blackburn, Dora Eliza Trinder, and Hannah Josephine Nolan, at the Shoreditch Union Infirmary.

Catherine Selina Selwood, at the Lambeth Infirmary.

Annie Elsie Vickery, at the Kensington Union Infirmary.

Eva Harriet Baker, Gertrude Olive Chadwick, Emily Winifred Connah, Emmie Dowling, Mary Matilda Griffiths, Louisa Ellen Haynes, Catherine Emily Lee, Florence Gertrude McFall, Isabella Parker, Margaret Potts, Hariette Alice Read, Agnes Rigby, Florence Lilian Sturman, and Alice Thompson, at the Liverpool Lying-in Hospital.

Teresa Maud Mary Walton and Violet Helen Wilson, at the Walton Workhouse, Liverpool.

Florence Wilhelmina Atkinson, Florence Gertrude Brown, Bertha Davies, Matilda Easterbrook, and Margaret Halliday Roddan, at the Liverpool Workhouse Hospital.

Caroline Catharine Böcker, Anna Hill, Adelaide House, Lily Annie Lawrence, Emily Louisa Parselle, Emily Jane Stephens, Alice Kate Waldrew, and Agnes Marguerite Wylie, at the Bristol Royal Infirmary.

Edith Marion Gauntlett, Constance Muriel Newton, Edith Ellen Perry, and Rena Emily Smith, at the Bristol General Hospital.

Annie Florence Angus, Dora Dransfield, Elizabeth McRae, and Frances Mary Pilkington, at the Jessop Hospital, Sheffield.

Sarah Janet Dockery and Lillie Cornelia Essen Edworthy, at the Nottingham Workhouse Infirmary.

Selina Harborough, Annie Louise Hawkins, Rebecca Ann Henry, Edith Isabell Hinde, Helen Thompson Husband, Muriel Florence Neison, Bessie Florence Potter, and Annie Smith, at the Brighton and Hove Hospital for Women.

Rachel Theodora Chatfield Croll-Dalgairns, at the Birmingham Workhouse Infirmary.

Harriett Cooke, Ellen Martin, and Lilian Maggie Routledge, at the Birkenhead Maternity Hospital.

Isabella Dawson, Benoy, Mary Ann Phillips, Laura Watson, and Mary Emily Sarah Young, at the Royal Derbyshire Nursing Association.

Hannah Fisher, Sarah Alice Ramsden, Mary Alice Pembroke, and Edith Mary Ward, at St. Mary's Hospital, Manchester.

Anna Biggs, Mary Graham, Anne Atkinson Hall, Marianne Sapcote Morrison, and Alice Robinson, at the Newcastle-on-Tyne Lying-in Hospital.

Jeannie Mulholland Adams, Margaret Conlon, Margaret Ann Dawson, Mary Matilda Knox, Madeline Naylor, Sarah Patton, Annie Margaret Rankin, and Mary Emily Walker, at the Belfast Union Maternity Hospital.

Bertha Susanna Collins, Maria Lassini Goodson, Ada Griffiths, and Elizabeth Monro, at the Queen Victoria Jubilee Institute for Nurses, Cardiff.

Mary Christabel Danson, at the Aberdeen Maternity Hospital.

Margaret Proctor Dawbarn, at the Hull Lying-in Charity.

Hélène Marie Doderet, Margaret Hawthorn, and Agnes Elizabeth Snowden, at the Louise Margaret Hospital.

Anne Dunn, trained at the Rotunda Hospital, Dublin.

Margaret Watson Eadie, Isabella Carr Ellwood, Sarah Elizabeth Lee, Mary Seater, Margaret Slattery, Violet Teale, Margaret Leslie Winchester, and Mary Winifred Youl at the Glasgow Maternity Hospital.

Thomasine Jane Eustice and Charlotte Elizabeth Lindsey, trained by the Gloucester District Nursing Society.

Amy Jane Felton, Mary James, Elizabeth Ann Paul, and Margaret Williams, at the Newport and Monmouthshire Hospital.

Kate Fern at the Greenwich Union Infirmary.

Janet Fraser and Mary Ellen Swindells, at the Dundee Maternity Hospital.

Lilian Keen Henshaw, Lucretia Hill, and Isabella Callow Stanbury, by the Cheltenham District Nursing Association.

Effie Ann James, Ethel Violet Lovell, and Eleanor Pridgeon, at the Cardiff Union Infirmary.

Edith Emily Smith, at the Coombe Lying-in Hospital, Dublin.

Winifred Frances Ingram, Mary Ann Lloyd, and Constance Emily Pracy, by the "Regions Beyond" Missionary Union.

Flora Susannah Smith, at the Ipswich Nurses' Home.

Adela Isabella Bode Austin, Elizabeth Annie Beattie, Mary Brennan, Winifred Alice Brierley, Mary Bright, Annie Budd, Kate Bune, Florence Annie Clift, Florence Cooper, Henrietta Sinclair Crawford, Jessie Frances Davenport, Elizabeth Kate Davies, Ellen Davies, Lilian Constance Duncan, Ethel Florence Farrow, Mary Bird Galloway, Sarah Gottman, Florence Mary Vansittart Graves, Emmeline Hackward, Mary Haldane, Nellie Higgins, Grace Margaret Hooper, Mary Ann Hosington, Charlotte Ann Houghton, Lilla May Howarth, Annie Jackson, Cecilia Marion Jackson, Naomi James, Elizabeth Martha Rose Johnson, Isabella Graham Johnson, Annie Helen Hamilton Jones, Catharine Jane Jones, Cecilia Evelyn Jones, Elizabeth McClymont, Clara Maylott, Mabel Willis Meachin, Mary Eveline Moore, Gertrude Amy Morley, Mary Hannah Muschamp, Harriet Elizabeth Olorenshaw, Edith Outram, Annie Catherine Owens, Mary Parker, Alice Mary Patchett, Violetta Ramsden, Emily Nora Ranger-Parton, Anne Reid, Jane Roberts, Janet Ewart St. Clair, Ellen Florence Sanders, Else Marie Schade, Sarah Scrimgeour, Beatrice Constance Mary Smith, Eleanor Stanton, Louisa Ellen Steadman, Lottie Swallow, Martina Taylor, Maria Payne Townsend, Alice Turner, Mary Watkins, Maria Antoinette White, Ella Widdows, Catherine Ann Wilson, Dora Elizabeth Wood, and Elizabeth Catherine Woods were trained by private individuals.

### Presentation to Miss Ramsden.

ON the occasion of her retirement from St. Marylebone Infirmary, Miss Ramsden was last week presented with a handsome pair of massive silver candlesticks and a silver compôte dish, bearing an appropriate inscription, by the medical and nursing staff of the infirmary.

Presentations were also made to her of an album containing all the names of the staff and the medical superintendent; a cut-glass claret jug, with silver top and handle, from the domestic staff; a silver bag-purse, from the resident medical officers; and a Russia leather card-case, from one of her late nurses now in Rome.

On the day previous to her departure last week a large number of nurses gathered together in the hall of her house and sang, in token of their esteem and affection, the hymn, "God be with you till we meet again."

It is with the deepest regret that the nursing staff of St. Marylebone Infirmary have said farewell to their dearly loved matron, and her place in their hearts will not easily be filled.

Miss Ramsden made each member of her staff, from the highest to the lowest, feel that she was not only their matron, but their friend, and it was as such that her nurses loved her and realised how much they have lost by her resignation.

She takes with her their best wishes for the future, and the hope that she may be rewarded somewhat for her labours among them by the knowledge that many young lives have been helped and encouraged at the beginning of their nursing career by her kindly interest and sympathy.

Miss Ramsden, who is now staying in Yorkshire, desires through our columns to thank her present and past nurses for their kind expression of sympathy for her on her retirement; and also all her hospital friends and nurses for the kind letters which she has received from them, all of which she has not yet been able to answer personally.

### Association for Promoting the Training and Supply of Midwives.

THE third annual meeting of the Association for Promoting the Training and Supply of Midwives was held on February 21 at 2 Cromwell Houses. Dr. Cullingworth was in the chair; and Mrs. Wallace Bruce gave the meeting a general outline of the report.

The report states that 551 applications for training have been received during the year, as against 433 in 1905. Of these 12 have been trained, as against 25 in the previous year. This reduction is partly due to want of funds; vacancies in the training schools have to be secured some months in advance, and the Council regret that several good candidates were lost owing to lack of funds and being unable to hold out hope of their being trained within a reasonable time. All the pupils were successful in passing the C.M.B. examination. The committee has been asked by the Nursing Associations of Shropshire, Somersetshire, and Gloucester to train women for work in those counties, in some cases sending up the pupils and paying towards their training. The Northamptonshire County Council has granted two annual scholarships for midwives, and the Association is to undertake the training of the women. Thirty-seven midwives who had been trained by the Association were now at work in different parts of the country, and all were reported to be doing excellent work.

The work at the Home continues to increase, the number of births attended this year being 864, as against 630 last year. The number of visits paid were 15,892 in 1906 and 12,283 in 1905. Mrs. Wallace Bruce and Miss Lucy Robinson both testified to the high standard both of training and moral influence which Nurse Rabson, the chief midwife, maintains, and to whom the success of the Home is largely due.

With the object of keeping in touch with the midwives, the Association has instituted a plan of giving a badge to those who have completed six months' satisfactory work in their districts.

An Advisory Committee has lately been formed, of which Mr. T. Almond Hind has consented to act as Chairman. Among the members of this committee are Dr. Champneys, Dr. Cullingworth, Miss Gibson, Miss Gill, Sir Shirley Murphy, Miss Paget, Miss Wilson, and many others, including several county medical officers. The committee is to inquire into and consider the whole question of the supply of midwives necessary to meet the requirements of the Act in 1910. According to statistics issued by the Central Midwives Board, there are 12,255 midwives practising in England and Wales, of whom 8,115 are untrained and enrolled under the *bona fide* clause of the Act. It is easily seen therefore the enormous deficit there will be in 1910, when these women "go out"—a serious question both to poor mothers and to Poor-law authorities, upon whom heavy expenses of medical attendance will fall unless measures are taken to fill up the gap.

Mr. Theodore Dodd spoke of the responsibilities of Boards of Guardians in the matter of relieving mothers and infants, and among other speakers were Mrs. Chas. Ebden, Miss Grant, and Mr. Leon.

### Queen Victoria's Jubilee Institute for Nurses.

MISS LUCRETIA HILL has been appointed to Cheltenham, Miss Rosa Lambert to Rawtenstall, and Miss C. E. Lindsey to Little Shelford; Miss Lillie Steele temporarily to Guildford, Miss Imelda Tegarty temporarily to Withington, and Miss E. N. Epps temporarily to Winslow. Miss E. C. Birch has been transferred to Matlock from the Liverpool Central Home.

### Nurses' Radius Agreements.

At Bournemouth last week a masseuse and nurse brought an action aginst the matron of the Dowsing Electrical Institute in that town for the sum of £6 15s. 8d., representing damages for an alleged breach of contract. Through the agency of an institution known as the Tabitha Training Nurses' Association the nurse had been engaged at the institute at a salary of £25 a year and a further sum of 15s. a week as remuneration for pupils and for attending to out-patients. It was also agreed that the nurse was to receive her fare from London to Bournemouth, and her return fare if she remained more than three months. She was told that she would be required to sign an agreement. A short time after her arrival on December 2 she was asked to sign an agreement that she would not carry on business nor act as a nurse within a radius of twenty miles of Bournemouth. With regard to acting as an electrical nurse, she was quite willing to sign this agreement, but refused to do so as to a general nurse. Ultimately she declined to sign the agreement at all, and left on December 22. The judge expressed his opinion that the agreement which the plaintiff was asked to sign went beyond the arrangement suggested at the time of her engagement, and he gave judgment for the nurse for the salary claimed, for the return fare from London to Bournemouth, and a certain sum for instruction to pupils and assistance to out-patients—namely, £5 0s. 8d. in all, together with the costs in the action.

### Appointments.

Coventry and Warwickshire Hospital, Coventry.—Miss Amy Lauder has been appointed night Sister. She was trained at the Royal Infirmary, Preston, where she was afterwards theatre charge nurse. She has since been Sister at the Royal Isle of Wight Infirmary and County Hospital, Ryde, and Sister at the Stockport Infirmary.

Gressenhall Infirmary, Norfolk.—Miss Nellie May Hitchcock has been appointed superintendent nurse. She was trained at Epsom Infirmary, and has since been staff nurse at Christchurch Infirmary and charge nurse at Epsom Infirmary. She holds the certificate of the Central Midwives Board.

Hartlepool Infectious Diseases Hospital.—Miss Edith Dawson has been appointed charge nurse. She was trained at the London Fever Hospital, and has since been staff nurse at the Isolation Hospital, Warrington, temporary nurse at Rothwell, Methley, and Hunslet Joint Isolation Hospital, and senior charge nurse at the Isolation Hospital, Burslem.

Haywards Heath Cottage Hospital.—Miss Lois E. Hulme has been appointed staff nurse. She was trained at Croydon Infirmary, and has been midwife at Plaistow Maternity and District Nursing Association.

Princess Alice Memorial Hospital, Eastbourne.—Miss Florence Perkins has been appointed senior Sister and Miss Katherine Boyle night Sister. Miss Perkins was trained at Mill Road Infirmary, Liverpool, where she has since held the post of Sister of wards for three years. Miss K. Boyle was trained at Mill Road Infirmary, Liverpool, where she has since held the post of Sister of a large medical ward.

Royal Bath Hospital, Harrogate.—Miss A. Parry has been appointed staff nurse. She was trained at the Salford Union Infirmary, Pendleton, Manchester.

Spittlesea Infectious Hospital, Luton.—Miss Olive Mallett has been appointed staff nurse. She was trained at the Isolation Hospital, Dartford, and has since been nurse at the Isolation Hospital, Ampthill.

### Death in our Ranks.

On Friday last, at the Royal Devon and Exeter Hospital, Sister Summerhayes (Miss Linda Chegwidden) passed away after suffering for several months from a painful malady. The funeral took place at Newquay on Wednesday this week, and simultaneously a memorial service arranged by the Chaplain was held in the hospital chapel.

### Everybody's Opinion.

### A PROBATIONER LOSES HER SIGHT.

"Nurse M.," Southend, writes: Will you please give the enclosed order for three shillings to the nurse who recently lost her eyesight while attending to a typhoid patient at West Ham Infirmary. She has my deepest sympathy.

### SUICIDE OF A PROBATIONER.

"D. Z. B." writes: Referring to the pathetic and interesting letter of Mr. Roberts in last week's issue as to the death of his niece, in which he appears very rightly and indignantly to deny that she poisoned herself, and further asserts that she could have had no reason for doing so, I would venture, if you have space, to say that not so very long ago, in a hospital that I think, for obvious reasons, I had better not name, a young lady whom I knew very well poisoned herself for no other reason than that she had been rather severely reproved by the matron. It is true that the girl I refer to had rather a violent temper, and could not bear being reproved in any way; but, in spite of her being in a properly conducted hospital, and herself a very young and inexperienced nurse, she certainly contrived to put an end to her life very successfully. I am the very last person to defend suicide, but I humbly venture to think that it is not always the unpardonable crime that society thinks it to be.

### THE DISTRICT NURSE AND THE PARISH DOCTOR.

"Kentish Nurse" writes: I can sympathise with "E. W." When I was working in Surrey I contracted typhoid fever. I sent twice for the doctor, waiting three days for him. The vicar was manager, and so his doctor was advised, but as he had a journey of six miles to make to get to me I frequently did not get necessary attention. During my work in my present district I had hæmatemesis. I asked two doctors' advice some time before, as I was in great pain, temperature 96°, pulse 50, and each doctor told me that many people have a subnormal temperature, and that there was nothing to be alarmed about; consequently I went on until I collapsed. The parish doctor being away on his holidays, I could not consult him; he is most kind, but his *locum* attended me until his return. In all, three doctors were telephoned for, but each answer was "Not at home," although the time of sending was 8 to 9 a.m. It was midday before I could get one. A doctor who has attended a nurse friend says he will not attend nurses gratis, and sent in the bill. If doctors do not care to attend district nurses, why do they not say so, instead of leaving us unattended? I think the best plan "E. W." can follow is to change her district. The nurse in the next parish to mine is working under the same difficulties, consequently she is retiring. I fail to see where the wrong comes in. Why should a nurse have a doctor she does not care for? She should be as free to choose as other individuals.

### Novelties for Nurses.

(By our Shopping Correspondent.)

### THE "SPINALIFE" BRUSH.

This is a useful and handy piece of apparatus, made by the Spinalife Brush Company, Carlton House, 11 Regent Street, S.W., and sold at 12s. 6d. The brush itself, of hog bristles stoutly inserted on a wire frame, will stand a good deal of rough usage, and as it is of simple construction it is unlikely to give trouble by getting out of order. Its uses are manifold. Thus it will prove of service in cases where skin-friction is essential, after the cold bath, etc., and it should be a valuable adjunct to the armamentarium of the masseuse. For self-use its usefulness will be increased by curving, or at any rate lengthening, the handle. As at present made the straight handle prevents the user from obtaining the maximum pressure when employing the brush for friction purposes.

### A Book and its Story.

### A SHADOWED LIFE.*

"God builds the nest of the blind bird," says the old proverb from which Mary Findlater takes the title of her pretty but rather sad little Devonshire story. Agnes Sorel, the heroine, stands for the character of one moving in the shadow of an unnamed sorrow, which, when its nature is made known to her, darkens her life and robs it of the joy natural to girlhood. The book begins as Agnes is leaving school and returning to live with her grandmother. The schoolmistress, half-aware of the mystery connected with her father's name, is full of sympathy for the girl. "Agnes said good-bye to Miss Brown, the young governess, and the schoolmistress; then, without a smile, not trusting herself to look back again at her companions, she went slowly across the hall, through the white-pillared porch that was set round with pots full of scarlet flowers, out into the sunshine. She entered the carriage, and it rolled away. They had a last glimpse of her white face, with deep unchildlike woe stamped upon it, as she looked farewell to the little world of school. 'Poor child! poor child!' said the schoolmistress. Miss Brown looked up with tears in her eyes. 'My old nurse used to tell me,' she said, 'that God builds the nest of the blind bird. Agnes is a "blind bird" if ever there was one.' She turned to speed another parting and said no more." Agnes, on arriving at the little country station, gets into the solitary fly waiting there, and is driven through a varying and ever-lovely country until it reaches the ferry which takes passengers over the river to the village of Ponde, where her grandmother lives. The author is particularly happy in the description of scenery, and brings the places described vividly before the reader. The village of Ponde as described is one of many other similar nooks in lovely Devon. "The village of Ponde lay on the side of the hill. So steep was the single street that it looked like a flight of stairs. Roads wound somewhere about on the heights above the houses; but the whole immediate life of the place was carried on by water. Buried, hidden, secret as it was from the rest of the world by land, the great waterway in front of it was its connection with life. The boats of the Romans had passed up there. Countless generations had come and gone, leaving no marks behind them. The small low-browed houses on the quay were the only successors of others more ancient, that had watched the broad highway with the eyes of their little windows for hundreds of years. It looked like a place to hide in, and then to forget and be forgotten. . . . The sun was going down behind the hill; already the houses by the quay were in shadow, and the line of the poles for the salmon nets showed white to the left as the boat came softly into the shore. No one had come to meet her; Agnes got out of the boat, paid the man, and turned, a lonely little figure, to walk up the precipitous street by herself." As Agnes draws nearer her destination, she pauses and takes in the scents and sounds that are borne on the evening air: the smell of salt water which, coming up with the tide, brings a freshness to the woods across the ferry from the open sea. From below she can hear the voices of men, and the sound of oars from a boat where Marines are training. "The sharp words of discipline shouted out by the officer in command—the flash of vigour and man's endeavour—gave a moment's life to the silent lane. She held up her head and walked briskly, as if the words of command had been addressed to her." The quaint house to which she is bound has been the home of her grandmother for some years. Here she has retired, and severed all connection with the outer world since the tragedy which darkened her life occurred. Agnes enters the narrow, dark-panelled hall, and is welcomed by an old maidservant, from whom she hears the unwelcome news that her aunt, "Miss Clare," has come home. She goes upstairs into her grandmother's sitting-room. "The last rays of sunset came glaring hotly in, striking full on the figure of a large old woman in a black dress, standing near the window leaning on a cane. . . . The old woman was largely built, still massive in figure. . . a woman once full of the force and pride of life; still, even in old age, very much alive." Her grandmother asks Agnes about her future plans, as it is decided she is not to return to school. Then she says that perhaps Agnes can continue to study with the help of her Aunt Clare, who is to be at home all the winter; but Agnes knowing that would not be possible says so. "Oh, Aunt Clare? She won't help me, you know. . . ." "There she is," said Agnes. A door is open, and through it comes the sound of a woman's voice between fits of shallow giggling laughter. This is followed by the entrance of a woman, overdressed, untidy, and indolent-looking. "Why, Nancy," she exclaimed, giving Agnes a hasty kiss, "is this you? You are just the same white-faced peaked-looking thing that you used to be. . . . Get out of that chair and give it to me, for I am just dead with fatigue." Her grandmother questions Agnes, and learns that at the breaking-up school dance among the guests who had been kind to her was a young American, Terence Woods, and his mother and Captain Bassett. "Ah, Mark Bassett?" Mrs. Sorel's tone changed instantly. She sat up and rubbed her hands across her eyes. "I haven't seen him for many years." . . . "He was quite kind," said the girl slowly. "He told me he had known my mother." She looks up as she says the last words in a loud tone, and her grandmother in reply tells her: "He was your father's friend." Her mouth gave a painful twitch as she spoke, and her hand closed on the head of her cane. "They were at College together. Austin and he were great friends." Later, when asking for some information about the parents she has never known, Agnes is told that her mother is dead, but that her father is alive. In answer to her question of where he is her grandmother replies: "Your father is not dead, Agnes." "'Oh, grannie! Where is he, then?' asked the girl. . . . 'Your father is in prison, child, the convict prison; you had better not speak to me any more about it, for I cannot bear it. . . . He killed a man in a moment of passion. There have been people more wicked than that!' Mrs. Sorel's lips twitched into a queer smile that frightened the child as she spoke." In this fact lies the elements of the tragic story. It is treated by the author in the simplest possible manner. By way of contrast nothing can be prettier than the relations of the American son, Terence Woods, and his mother. The mutual devotion is typical of the new country, and it makes delightful reading. Mrs. Woods, with her good looks, sweet manners, and perfect dressing, comes into Agnes Sorel's life as a vision of beauty from another world and the frank friendship of Terence and Agnes ripening into romance bears the test of time and trial and brightens the background of her sad life. Readers will find in "A Blind Bird's Nest" much that is charming and nothing to shock the most fastidious.

* "A Blind Bird's Nest." By Mary Findlater. (Methuen. 6s.)

### Notes and Queries.

**REGULATIONS.**

**The Editor is always willing to answer in this column, withou any fee, all reasonable questions, as soon as possible.**

**But the following rules must be carefully observed.**

**1. Every communication must be accompanied by the name and address of the writer.**

**2. The question must always bear upon nursing, directly or indirectly.**

**If an answer is required by letter a fee of half-a-crown must be enclosed with the note containing the inquiry.**

*Rheumatic Arthritis.*

(217) Please inform me where a lady suffering from rheumatoid arthritis can be received and have the Salisbury diet.—*A Friend.*

Any good home for invalids would provide the Salisbury diet at request. You will find a list in "Medical Homes for Private Patients," price 7d. post free, The Scientific Press, 28 and 29 Southampton Street, Strand, London, W.C.

*Probationer.*

(218) I wish to become a nurse, but have been refused at two hospitals, as I am only 18; where can I be received with a small salary?—*Kate.*

No general hospital training school will receive you at 18. If you wish to be qualified for a good post in the future you must wait until you are 23, or you might be received in some children's hospital a little younger. The Alexandra Hospital for Children with Hip Diseases, Queen Square, Bloomsbury, W.C., is open to candidates of 18 and upwards. Get "How to Become a Nurse," price 2s. 4d. post free, The Scientific Press, 28 and 29 Southampton Street, Strand, London, W.C.

*C.M.B.*

(219) I am a trained nurse, and am anxious to obtain the C.M.B. I cannot afford to pay a premium. Can you help me to free training?—*Woking.*

Free training is almost impossible to obtain, but write to the Association for Promoting the Training and Supply of Midwives, Dacre House, Dean Farrar Street, S.W.; or the Rural Midwives Association, 47 Victoria Street, S.W.; possibly, too, the Ladies' Benevolent Fund, 7 Grosvenor Street, Chester, might help you. An advertisement might possibly bring you a satisfactory answer.

*Infirmary Training.*

(220) Can you tell me of a general hospital where probationers with Poor-law nursing experience would be received?—*Jim.*

Get "How to Become a Nurse," price 2s. 4d. post free, The Scientific Press, 28 and 29 Southampton Street, Strand, London, W.C., and write perseveringly to the matrons of any hospitals you may select.

*Nottingham Children's Hospital.*

(221) If I enter as probationer at this hospital shall I get a salary to start with?—*Clara.*

Yes, £5 first year, £10 second year, £12 third year. But you must enter for the three-years' training.

*Nursing in Rome.*

(222) Will you tell me the name of a nursing home in Rome?—*Brighton.*

The Anglo-American Nursing Home, 265 Via Nomentana. See our rules relating to answers to correspondents.

*C.M.B.*

(223) Which is the cheapest way to train for the C.M.B. certificate? Also, does the place of training make any difference to the chance of getting work afterwards? Is there any special place where a nurse can borrow money for training, paying it back gradually afterwards? I have had three years' training, so I do not know whether I should get more help than a person with none at all.—*Derbyshire.*

See answer to *Woking*, also write for advice to the Midwives' Institute, 12 Buckingham Street, Strand. Enclose a penny stamp.

**Handbooks for Nurses.**

| | Post Free. |
|---|---|
| "How to Become a Nurse: How and Where to Train." | 2s. 4d. |
| "Nursing: its Theory and Practice." (Lewis.) ... | 3s. 6d. |
| "Complete Handbook of Midwifery." (Watson.) ... | 6s. 4d. |
| "Preparation for Operation in Private Houses." ... | 0s. 6d. |
| "The Nurses' Enquire Within." ... ... ... | 2s. 3d. |
| "Nurses' Pronouncing Dictionary of Medical Terms" | 2s. 0d. |

Of all booksellers or of The Scientific Press, Limited, 28 & 29 Southampton Street, Strand, London, W.C.

### For Reading to the Sick.

STRENGTH IN WEAKNESS.

When I am feeble as a child,
And flesh and heart give way,
Then on Thine everlasting strength
With passive trust I stay,
And the rough wind becomes a song,
The darkness shines like day.

Deep unto deep may call, but I
With peaceful heart will say—
"Thy loving-kindness hath a charge
No waves can take away:
And let the storm that speeds me Home
Deal with me as it may."

*A. L. Waring.*

Let us take to ourselves these comfortable thoughts, both in the contemplation of our own death or upon the death of our friends. Wherever faith in Christ is, there is Christ Himself. He said to Martha, "Believest thou this?" Wherever there is a heart to answer "Lord, I believe," there Christ is present. There our Lord vouchsafes to stand, though unseen—whether over the bed of death, or over the grave: whether we ourselves are sinking, or those who are dear to us. Blessed be His Name! nothing can rob us of this consolation: we will be as certain, through His grace, that He is standing over us in love, as though we saw Him. We will not, after our experience of Lazarus' history, doubt an instant that He is thoughtful about us.—*J. H. N.*

There are secrets with which no stranger can intermeddle. When Mary of Bethany started forth from out of the chamber of sorrow, no one knew whither she was going, except her sister. Others thought she had gone to weep by her brother's grave. No eye but of One could know what secret spring was moving her. The idea which changed so suddenly the whole bearing and aspect, as she went forth at the Master's call, to cast herself on His loving support, was to her the more precious because it was secret. All Mary's history proves that hers was a specially hidden life. And we must bear in mind that she did not know Jesus as you know Him. Hers was a very limited knowledge, when contrasted with yours, yet even within that narrower sphere of thought she attained a complete restfulness in the loving union of her soul with His. How then will it be with you who know Him so fully, so unreservedly, in all His completeness of Sacrifice and Atoning Love, and Intercessory Power, if such assurances should not lead to a greater perfectness of loving union with Him?

. . . Yea, Lord Jesus, be it so. May Thy call reach the hearts of Thy children, especially in their times of need, of inner trial, of secret doubts. May they ever, as they hear Thy call, have power to arise and cast themselves into those Everlasting Arms, on that tender Heart; into the depths of that true love, and be sustained there even to the end.—*T. T. Carter.*

Jesus calls us; o'er the tumult
Of our life's wild, restless sea;
Day by day His sweet voice soundeth
Saying, "Christian, follow Me!"
Jesus calls us; by Thy mercies,
Saviour, make us hear Thy call.

*Hymn* 403 (*A. & M.*).

## Figures and Tables

**Figure f1:**